# Mediating roles of meaning in life and psychological flexibility in the relationships between occupational stress and job satisfaction, job performance, and psychological distress in teachers

**DOI:** 10.3389/fpsyg.2024.1349726

**Published:** 2024-05-03

**Authors:** Murat Yildirim, Ümit Dilekçi, Abdullah Manap

**Affiliations:** ^1^Department of Psychology, Faculty of Science and Letters, Ağrı İbrahim Çeçen University, Agri, Türkiye; ^2^Department of Social and Educational Sciences, Lebanese American University, Beirut, Lebanon; ^3^Department of Child Development, Faculty of Health Sciences, Batman University, Batman, Türkiye; ^4^Department of Psychology, Faculty of Arts and Sciences, Batman University, Batman, Türkiye

**Keywords:** meaning in life, psychological flexibility, occupational stress, job satisfaction, job performance, psychological distress

## Abstract

Employees may experience stress in the workplace for various reasons. Psychological strengths may help them to cope with emerging challenges and foster mental health and work productivity. This study examined the mediating roles of meaning in life and psychological flexibility in the relationships between perceived occupational stress and job satisfaction, job performance and psychological distress. This cross-section study included 554 teachers (56.0% males; *M*_age_ = 36.99 ± 7.88 years) at all school levels in Türkiye. The research data was collected through a convenience sampling method using an online survey. Participants completed self-report measures of meaning in life, psychological flexibility, perceived occupational stress, job satisfaction, job performance, and psychological distress. Pearson product–moment correlation and parallel mediation model using PROCESS macro (Model 4) were utilized to analyze the data. The results showed that perceived occupational stress had a significant effect on meaning in life, psychological flexibility, job satisfaction, job performance, and psychological distress. Also, meaning in life and psychological flexibility had significant effects on job satisfaction, job performance, and psychological distress. Furthermore, meaning in life and psychological flexibility partially mediated the relationships between occupational stress and job satisfaction, job performance, and psychological distress. These findings highlight the significance of considering meaning in life and psychological flexibility as crucial factors in mitigating the impact of occupational stress on employee mental health and work productivity. By focusing on enhancing employees’ sense of meaning and their ability to adapt flexibly to workplace challenges, organizations can potentially create a better environment that fosters positive outcomes for both employees and the organization. However, the impact of occupational stress on job performance, job satisfaction and psychological distress may change over time. To address this concern, future research should test the model through a longitudinal study design.

## Introduction

Along with the technological advancements of the twentieth century, newly-emerging working patterns and conditions have led people to assume different and individualized responsibilities and to face the expectation of ongoing job performance staying ahead of defined job descriptions (Savino, 2009). Accordingly, there has been a shift in the meaning attributed to work life by societies and individuals in such a working environment where individuality comes to the limelight. Individuals may attach importance to their roles in work life at least as much as their roles in family or social life. The developments in the fields of work life and psychology in the last two centuries and the relations between both concepts have aroused interest among many theorists and researchers. Thus, psychological factors such as occupational burnout (Ahola et al., 2013), professional incompetence (Behrens-Wittenberg and Wedegaertner, 2023), occupational stress (Mayer and Oosthuizen, 2021), and job dissatisfaction (Lambert et al., 2020) have emerged as important factors in the context of the work environment.

The present study aims to examine the effect of occupational stress on job performance, job satisfaction and psychological distress, as well as mediating the impacts of meaning in life and psychological flexibility, which are deemed as the factors preventing or reducing these negative effects. Occupational stress is linked with various physical, mental, emotional, and behavioral changes (Mayer and Oosthuizen, 2021) by possessing negative consequences for employers (Dolan, 2007; Yıldırım et al., 2023). Research highlighted that occupational stress has also increased costs and remained on the agenda of work life to a great extent (Siu et al., 2020). This may lead to a reduction in job performance and satisfaction which is an undesirable situation for both the employee and the employer. In addition to adversely affecting job performance and job satisfaction, occupational stress is also associated with the emergence of depression symptoms, which are an important source of psychological distress. Hong et al. (2022) highlighted that long working hours are related to occupational stress and occupational stress is associated with the symptoms of depression. Research showed that occupational stress resulting from low wages, irregular life, and lack of skills is associated with suicidal thoughts as well as depression symptoms (Mohamed et al., 2023). It has been acknowledged that psychological problems such as depression also reduce productivity in work life (Evans-Lacko and Knapp, 2016). There is a body of literature indicating that occupational stress is related to the home environment (Belwal et al., 2023).

Psychological stress caused by occupational stress may not affect everyone in the same way. The level of job satisfaction, poor job performance and psychological problems yielded by occupational stress can vary according to internal or external factors. To exemplify, Jung et al. (2023) concluded that positive emotions such as courage and perseverance reduce burnout, a symptom of depression arising from occupational stress. Accumulating evidence in the literature provides support for the increased significance of occupational stress both individually and organizationally (Dolan, 2007).

### Occupational stress

The term stress refers to the consequence of the meanings attributed to situations that exceed or deplete one’s resources, or to environmental loads which increase the risk of depletion (Lazarus and Folkman, 1984). The resources expressed by Lazarus and Folkman (1984) are related to individuals’ potential regarding their strategies for coping with stress and social support. Some experiences can adversely affect individuals’ coping skills and increase the effect of stress on the individual. For instance, when an individual with a high capacity to deal with a stressful problem in work life loses a relative, his/her coping skills may become dysfunctional. In this case, the individual is faced with a problem that exceeds his/her resources. Stressors can differ according to the roles in which we undertake a course of responsibilities. Therefore, the concept of occupational stress has been narrowed down compared to the term stress. The teachers’ job demands may not align with their abilities, available resources, or the needs of their colleagues. This situation is characterized by adverse physical and emotional reactions, leading to occupational stress (Schill and Chosewood, 2013).

Occupational stress is deemed as a psychological manifestation arising from negative emotional, mental, or behavioral effects such as strain, feeling under pressure and distress caused by challenging conditions and hob requirements encountered in work life (Lachowska et al., 2018; Mayer and Oosthuizen, 2021). The term has been defined in line with the hardships faced by people in work life along with the development of modern industrialization and the business world. The *European Agency for Safety and Health at Work* defines work-related stress based on work-related reasons (Brun, 2007) and it has been stated that stress occurs when job demands are not commensurate with the employees’ abilities, resources or needs of the worker (Lesage and Berjot, 2011). Foti et al. (2023) have analyzed occupational stress through the following dimensions: supervisor support, colleague support, job demands, job control, and role ambiguity. It can be said that the concept of job demand refers to the physical, mental, emotional or social difficulties that employees encounter during their work.

To illustrate, based on the demand-control model, it is argued that high job demand and low job control/support increase occupational stress (Karasek and Theorell, 1990). According to De Almeida Oliveira et al. (2023), occupational stress can stem from the overwhelming increase in duties and responsibilities, often accompanied by insufficient time to fulfill them. Additionally, it may arise from the cumulative demands placed on employees without corresponding rewards or recognition for their performance. Occupational stress can be characterized as the tension that arises from disparities between the expectations of employers or customers and the expectations of employees.

### Occupational stress and psychological distress

Stress can disrupt emotional balance and lead a person to feel more unhappy, hopeless, or helpless. This state of emotional strain triggers depression and anxiety and can increase the level of emotional damage (Khade et al., 2022). Correspondingly, it can be alleged that occupational stress increases psychological distress (Stansfeld and Candy, 2006). High levels of occupational stress have an adverse effect on job performance (Anbazhagan and Selvan, 2022) and job satisfaction (Kong et al., 2020) in addition to increasing psychological distress (Nishihara et al., 2022). Early studies have shown that such factors as strong workplace competition (Wang et al., 2017), job demands (Sorour and Abd El-Maksoud, 2012), excessive workload (Panhwar et al., 2019), and working hours (Adriaenssens et al., 2011) can be said to trigger the level of occupational stress. High levels of occupational stress may negatively affect job satisfaction (Kong et al., 2020) and job performance (Dolan, 2007). For instance, occupational stress has been linked to various health and wellbeing issues. It has been associated with physical symptoms such as headaches, fatigue, tachycardia, digestive and appetite problems, and insomnia (Metlaine et al., 2005; Khade et al., 2022). It can lead to emotional symptoms like anxiety, anger, irritability, and impact job satisfaction (Rožman et al., 2019). Additionally, it may result in cognitive problems such as difficulties with attention, concentration, and decision-making (Gutshall et al., 2017). Furthermore, occupational stress can contribute to behavioral problems such as reduced job performance, social withdrawal, and potentially even substance addiction (Frone, 2006). These findings collectively highlight that occupational stress is a widespread, significant, and costly issue in workplace health (Siu et al., 2020).

### Job performance and job satisfaction

Job performance is typically defined as the employees’ capacity to accomplish their duties and responsibilities and their work-related abilities (Campbell, 1990). Job performance is not a one-dimensional term (Ramos-Villagrasa et al., 2019), yet it is a multifaceted concept encompassing various elements such as technical knowledge and skills, communication skills, teamwork, problem-solving skills (Morgeson and Humphrey, 2006; Grant et al., 2009; Limon and Sezgin-Nartgün, 2020). Besides, the term refers to the individual’s contributions to the workplace to achieve organizational objectives (LePine et al., 2005; Campbell and Brenton, 2015). Work performance plays as one of the crucial factors in monitoring, offering feedback, and fostering development (Pulakos et al., 2015). Job performance is also associated with many factors such as employee self-confidence, manager support, authentic leadership, and empowering leadership (Limon, 2022; Zeb et al., 2023).

The concept of job satisfaction includes an individual’s affective, cognitive or behavioral responses to one’s job (Judge et al., 2017). Job satisfaction is a significant factor influencing employees’ happiness and contentment in their workplace (Luthans, 2011; Skaalvik and Skaalvik, 2011; Limon et al., 2021). A satisfying work life is associated with such important facets as employee motivation, job performance, job involvement, perception of autonomy and effective task performance (Riketta, 2008; Judge et al., 2017; Dilekçi, 2022). Various factors such as workplace culture, career opportunities, safety, wages, managerial attitudes and the alignment between the employee’s work and his/her values are the factors that represent job satisfaction (Erdogan and Enders, 2007; Erdogan and Bauer, 2009). Job satisfaction does not only depend on the conditions of the workplace but can also be related to the employee’s psychological state, authentic leadership (Zeb et al., 2020; Rehman and Zeb, 2023), personality traits, values and expectations (Weiss, 2002; Judge et al., 2017).

The findings in the literature confirm that occupational stress, an important psychological problem in work life, has a negative impact on job performance (Wright and Cropanzano, 2004; Sonnentag and Frese, 2013) and job satisfaction (Pindek et al., 2021). Podsakoff et al. (2007) noted that occupational stress-induced poor job satisfaction. In the same vein, Bakker and Costa (2014) reported that occupational stress negatively affected job performance. Unlike occupational stress, there are also findings in the literature supporting that both psychological flexibility and meaning in life enhance job performance and job satisfaction. Previous studies have shown that individuals with high levels of psychological flexibility (Hayes et al., 2012) and the ones with a positive perception of meaning in life (Steger et al., 2006; Martela and Steger, 2016) may also have high levels of job satisfaction. Furthermore, it has been acknowledged that psychological flexibility and the perception of meaning in life also influence job performance (Chalofsky and Krishna, 2009). Taken together, it has been seen that job performance and job satisfaction do not increase or decrease by themselves and that there are certain factors affecting both concepts either positively or negatively.

### Psychological flexibility and meaning in life as mediators

Psychological flexibility is defined as the ability to succinctly pursue valued life aims despite the presence of ever-changing demands of life while maintaining mental health (Hayes et al., 2006; Kashdan and Rottenberg, 2010). It refers to maintaining self-control and avoiding dysfunctional attempts as fully adopting and experiencing one’s feelings, thoughts, and sensations (Luoma et al., 2007). Acting with momentary feelings and thoughts can be considered as an example of dysfunctional attempts (Hayes et al., 2012). The term involves the acceptance of experiences associated with discomfort during valued goal pursuit (Hayes et al., 2006). It can be asserted that an individual’s accepting negative experiences improves his/her psychological flexibility. The concept of psychological flexibility includes the processes of acceptance, present moment, defusion, self-as-a-content, values, and committed action (Hayes et al., 2012). This term has recently been given great importance by researchers since it is closely associated with minimizing anxiety and depression (Kashdan and Rottenberg, 2010; Gloster et al., 2017) and increasing resilience against psychological distress (Luoma et al., 2007).

The meaning in life represents the individual’s perspective on existential aims and significance (Steger et al., 2006; Yıldırım et al., 2021) and has a significant effect on the wellbeing and mental health of individuals (Arslan et al., 2021; Arslan and Yıldırım, 2021). According to Martela and Steger (2016), meaning in life is the identification of personal values and objectives that provide a coherent sense of purpose and worthiness. Personal values and individual objectives can be posited as integral components of one’s professional life. As posited by Ning and Zhao (2016), the construct of meaning in life plays an important role in influencing job performance. It has been pointed out that psychological flexibility which enhances resilience and, therefore, has positive contributions to mental health, is closely linked to the meaning in life (Bohlmeijer et al., 2011). The relationship between psychological flexibility and meaning in life is also elucidated by setting meaningful goals and experiencing living for a meaningful purpose in life. Psychologically flexible individuals are more likely to engage in certain activities that align with their values, pursue meaningful goals, and experience a greater sense of purpose and coherence (Kashdan and McKnight, 2013).

Over the past couple of decades, the positive psychology approach has been thought to have put forward an important perspective to overcome many psychological problems. The concepts of meaning in life and psychological flexibility are the two main facets of positive psychology. Quan et al. (2022) found an opposite directional relationship between psychological flexibility and psychological distress in the workplace. It was also revealed by another study that there were significant relationships between job satisfaction and burnout and psychological flexibility and that psychological flexibility had an effect on mental flexibility and psychological distress (Chong et al., 2023). In spite of increasing rigor in positive psychology, little has been said about the concepts of meaning in life and psychological flexibility concerning the problems related to teachers’ professional lives.

Occupational stress may suppress job satisfaction (De Simone et al., 2016; Kong et al., 2020; Ngirande, 2021) and job performance (LePine et al., 2005; Dolan, 2007; Anbazhagan and Selvan, 2022). Indeed, occupational stress may be the source underpinning psychological distress (Hasan and Tumah, 2019; Ta’an et al., 2020; Nishihara et al., 2022). The relationship between stress and psychological distress may suggest that stress is more likely to negatively affect psychological resilience since excessive or continuous stress can adversely affect a person’s emotional functions and behaviors. Allan et al. (2016) found a negative relationship between occupational stress and meaning in life. On the contrary, the greater levels of such concepts that positively affect mental health as the meaning in life and psychological flexibility can contribute to minimizing distress and fostering job satisfaction and job performance. In light of the preceding findings, the present study is devoted to examining the mediating roles of meaning in life and psychological flexibility in the relationships between occupational stress and job satisfaction, job performance and psychological distress.

### Present study

The challenges of work-life are regarded as an important factor influencing individuals’ wellbeing and mental health. Occupational stress can sometimes be the source of these hardships or the outcome of those difficulties in the workplace. Moreover, there is an accumulating body of literature suggesting that occupational stress has significant negative consequences. Early studies have shown that a high level of occupational stress reduces job satisfaction and job performance while increasing psychological distress (Podsakoff et al., 2007; Bakker and Demerouti, 2017).

Unlike negative factors, positive psychological constructs are known to have positive effects on mental health and wellbeing (Yildirim and Belen, 2018; Yildirim et al., 2019; Masoom Ali et al., 2020; Green and Yıldırım, 2022; Rehman et al., 2023; Yıldırım et al., 2024). The related body of literature on psychological flexibility and meaning in life has demonstrated that the emphasis is placed on the aspect of mental health improvement (Quan et al., 2022; Chong et al., 2023). In addition, it can be said that they also contribute to daily life, work life or family life. The fact that the mediating roles of meaning in life and psychological flexibility in the relationships between such personal psychological experiences as occupational stress and job satisfaction, job performance and psychological distress are investigated is of great importance in terms of guiding individuals on how they can enhance job performance and job satisfaction and how they can protect themselves from discomfort in the face of the negative effects of occupational stress. To this end, the present study was designed to provide insight into the mediating roles of meaning in life and psychological flexibility in the relationships between occupational stress and job satisfaction, job performance and psychological distress. Addressing this aim, the following hypotheses are formulated.

*The first hypothesis* is that occupational stress has a direct effect on psychological flexibility, meaning in life, job performance, job satisfaction and psychological distress. The existing literature indicates that occupational stress reduces job performance and job satisfaction (Sonnentag and Frese, 2013) and that it triggers certain psychological problems such as depression, anxiety, anger and pessimism (Rožman et al., 2019; Nishihara et al., 2022) and that it has negative effects on psychological flexibility and the meaning in life which have protective roles on mental health (Nishihara et al., 2022).

*The second hypothesis* to consider is that psychological flexibility exerts a direct influence on job performance, job satisfaction, and psychological distress. Psychological flexibility equips individuals with enhanced coping mechanisms to effectively deal with stressors, ultimately leading to reduced distress, improved adaptation, and increased job performance (Bond et al., 2008).

*The third hypothesis is*, however, that the meaning in life has a direct effect on job performance, job satisfaction and psychological distress. Evidence in the literature provides support for individuals with a positive perception toward the meaning of life may also have high levels of job satisfaction and job performance (Steger et al., 2006; Chalofsky and Krishna, 2009; Martela and Steger, 2016). Eisenbeck et al. (2022) claimed that depression and anxiety levels decreased with the increasing level of meaning in life.

**Figure 1:**
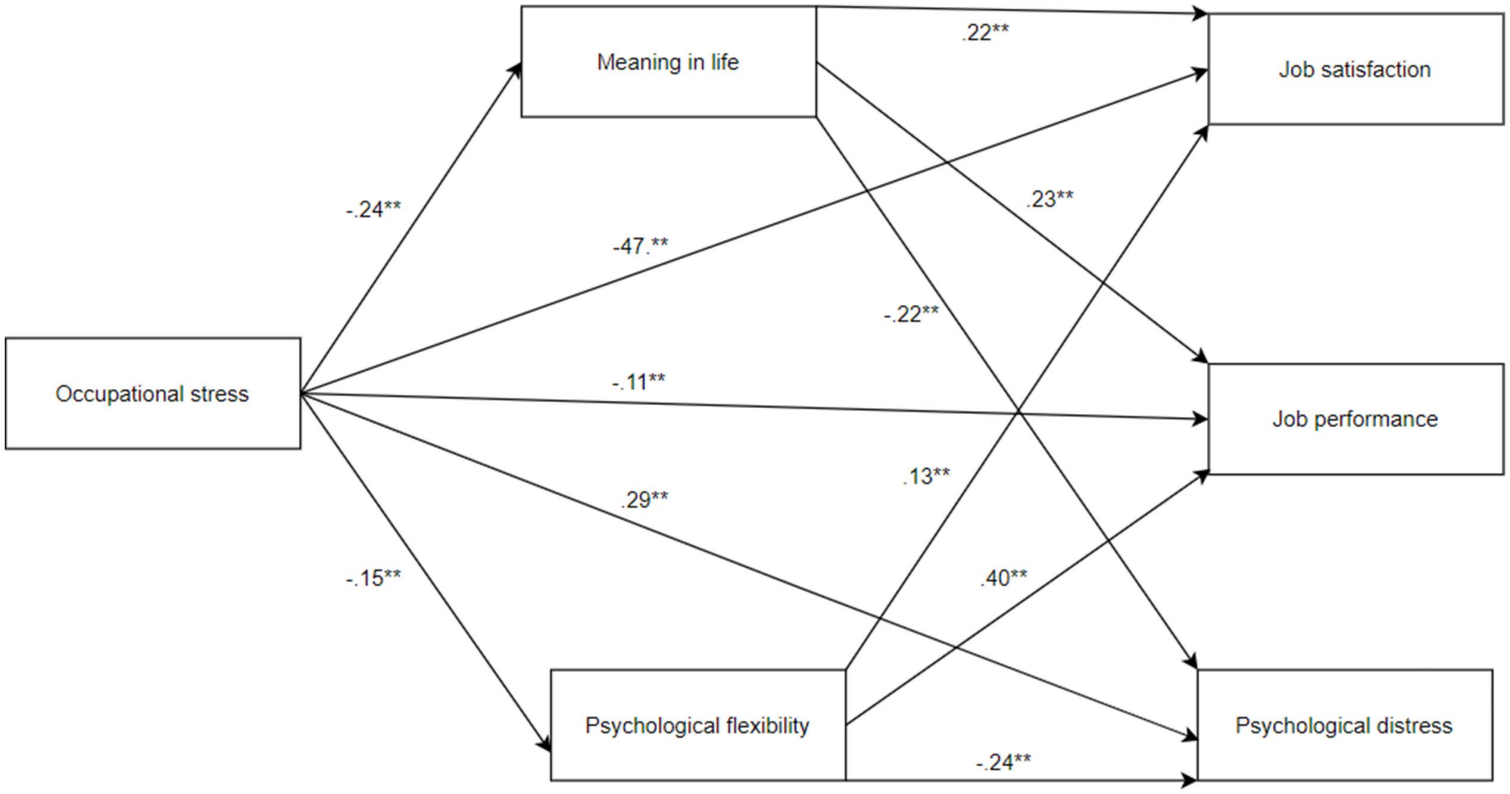
Conceptual model of the hypothesized associations among the study variables. The values presented in the figure represent the standardized coefficient values. **p < 0.01.

*The fourth* is that the effect of occupational stress on job performance, job satisfaction and psychological distress is mediated by psychological flexibility and the meaning in life. Psychological flexibility and meaningful goals that are related to the meaning in life have been identified as potential protective components against certain negative situations such as stress and growing up unsuccessfully (Boyle et al., 2015). Stress is an inherent aspect of human existence, and its complete elimination is not a realistic objective. Rather, the emphasis lies in cultivating the capacity to effectively manage stress during challenging circumstances or possessing psychological attributes that safeguard against the deleterious impacts of excessive stress, which can culminate in enduring harm to one’s mental wellbeing. Consequently, this study seeks to augment existing literature by elucidating the factors that mitigate the adverse influence of stress on individuals, specifically pertaining to job performance, job satisfaction, and psychological wellbeing. Within this context, elevated levels of psychological flexibility and a robust sense of life’s positive meaning are postulated to exert a protective influence on mental health outcomes. It has been found that individuals who deal with stressful situations in a more meaningful way have better stress management skills and greater success in coping with psychological distress (Park, 2010). Accordingly, it is assumed that the meaning in life and psychological flexibility will mediate the relationships between occupational stress and job satisfaction, job performance and psychological distress as well as minimizing the negative effects of stress on these consequences. Figure 1 displays the hypothetical model for the relationships between variables.

## Methods

### Participants

Participants were selected using a convenience sampling approach, which is a non-probabilistic sampling technique widely used in studies where the primary focus is on practicality and accessibility. In our case, this approach was chosen due to its suitability for collecting data from a large and diverse pool of potential participants. A total of 554 teachers at all school levels, including preschool, primary school, middle school, and high school in Türkiye took part in this study. The participants consisted of 56.0% males and 44.0% females, with the majority reported being married (75.3%), followed by single (23.8%) and widows or widowers (0.9%). The age range of the participants varied between 20 and 65 years, with an average age of 36.99 years (*SD* = 7.88). The professional experience of the participants varied, with the majority having 0–5 years of experience (23.6%), followed by those with 6–10 years (22.0%), 11–15 years (16.8%), 16–20 years (17.5%), and 21 years and above (20.0%). Also, the participants in the study had diverse professional experience in the education sector, with the majority having experience in secondary schools (39.5%), followed by primary schools (31.4%), high schools (24.2%), and preschools (4.9%). With regard to the highest level of education, a substantial percentage of participants had a Bachelor’s degree (82.7%), while a smaller portion held a Master’s degree (15.5%), and a PhD degree (1.8%).

### Measures

*Perceived occupational stress* (POS; Marcatto et al., 2022). The POS consists of four items designed to assess workers’ perception of occupational stress experienced in the previous 6 months. Participants rate their level of stress in the workplace on a 5-point Likert scale, ranging from 1 (strongly disagree) to 5 (strongly agree). The total score across the four items is calculated to determine the overall POS score, which can range from 4 (indicating the lowest perceived stress) to 20 (indicating the highest perceived stress). The Turkish validation of the POS was conducted by Yıldırım et al. (2023) who showed good evidence of reliability and validity. They confirmed the unidimensionality of the scale and reported high internal consistency reliability in the Turkish language. In the present study, Cronbach’s alpha was found to be 0.85.

*Index of job satisfaction* (Brayfield and Rothe, 1951) is a self-report instrument created to assess job satisfaction. The short version of the scale comprises five items, with participants indicating their agreement on a five-point scale ranging from 1 (strongly disagree) to 5 (strongly agree). The scale has demonstrated satisfactory reliability (e.g., internal consistency reliability) and validity (e.g., construct validity) evidence in the Turkish context (Keser and Bilir, 2019). In this study, Cronbach’s alpha was found to be 0.85.

*Job performance scale* (JPS: Çalışkan and Köroğlu, 2022) was utilized to assess the job performance of employees in this study. The JPS comprises 11 items, and participants indicated their responses on a 5-point Likert-type scale ranging from 1 (strongly disagree) to 5 (strongly agree). The scale encompasses two subscales, namely task performance and contextual performance. In the context of this study, a total score was calculated, where higher scores on the JPS reflect elevated levels of job performance. The JPS demonstrated good psychometric properties with high evidence of construct validity, discriminant validity, criterion validity, and internal consistency reliability with Turkish employees (Çalışkan and Köroğlu, 2022). Cronbach’s alpha coefficient was found to be 0.91 in this study.

*Kessler Psychological Distress Scale* (K6; Kessler et al., 2002) was used to evaluate symptoms of psychological distress experienced in the past 30 days. This scale includes six items, and participants rated their experiences on a 5-point Likert-type scale ranging from 1 (none of the time) to 5 (all of the time). Higher scores on the K6 indicate a greater level of psychological distress. Previous research conducted with Turkish adults has demonstrated sound psychometric properties (e.g., inter-item correlation, internal consistency reliability, and construct validity) for the K6 (Altun et al., 2019). Cronbach’s alpha was found to be 0.78 in the current study.

*Meaning in Life Questionnaire—Short Form* (MLQ-SF; Steger and Samman, 2012) was used to assess one’s overall meaning in life. The MLQ-SF consists of three items, and participants rated each item on a 7-point Likert scale, ranging from 1 (absolutely untrue) to 7 (absolutely true). Higher scores on the questionnaire indicate a higher level of meaning in life. The Turkish version of the original Meaning in Life Questionnaire was translated by Demirbas (2010). In the present study, Cronbach’s alpha was found to be 0.84.

*Psy-Flex Scale* (Gloster et al., 2021) is a self-reported measure designed to assess contextually sensitive psychological flexibility. This unidimensional scale consists of six items, and participants rate each item on a 5-point Likert scale, ranging from 1 (very seldom) to 5 (very often). The Turkish validation of the scale was conducted by Yildirim and Aziz (2023), who provided strong evidence regarding the validity (i.e., construct validity) and reliability (i.e., internal consistency reliability) of the scale in the Turkish context. To calculate the total score, all item responses are summed, with higher scores indicating a higher level of psychological flexibility. Cronbach’s alpha was found to be 0.72 in the current study.

### Procedure

For the current study, a web-based survey was created, consisting of an information sheet, demographic questions, and primary questionnaires. The survey was disseminated through a secure online platform. To maximize the diversity of the sample, the survey link was shared on various social networking sites such as Twitter (X), Facebook and WhatsApp. Prior to their involvement in the study, participants were provided with clear instructions highlighting the confidentiality and anonymity of their responses. All participants provided their informed consent before participating in the study. The questionnaires were presented to participants in a consistent order. Participation in the study was voluntary, and participants did not receive any form of compensation for their involvement. Our study did not have any missing data as all participants were required to answer all questions. Participants who chose not to complete all items opted out of the survey, resulting in complete data records. The research protocol received approval from the Ethics Committee of Batman University in Türkiye (2023/05–07), thereby confirming adherence to the ethical principles delineated in the 1964 Helsinki Declaration.

### Data analysis

The sufficiency of sample size serves a crucial role in detecting any effects between the variables. In this study, a sample size of 554 participants was used, which was greater than the recommended range of 115–285 participants suggested for mediation analysis to identify indirect effects among the variables (Fritz and MacKinnon, 2007). Prior to conducting the main analysis, descriptive statistics including mean and standard deviation statistics were performed to examine the characteristics of the scales and the assumption of normality for the study variables. Normality was assessed through an examination of kurtosis and skewness scores, as presented in the Results section. These analyses confirmed that the data followed a normal distribution, thereby affirming the appropriateness of employing parametric tests in this study. Subsequently, a Pearson product–moment correlation analysis was conducted to explore the associations between the study variables. Afterwards, the proposed parallel mediation model was tested using the PROCESS macro (Model 4) for SPSS version 26 (Hayes, 2018). The PROCESS macro (Model 4) was chosen for this analysis because it is specifically designed for examining parallel mediation models, which involve multiple mediators operating simultaneously. In our study, we aimed to investigate how meaning in life and psychological flexibility collectively influenced the relationship between occupational stress and job satisfaction, job performance, and psychological distress. PROCESS Model 4 allows us to assess these indirect effects concurrently and estimate their significance, making it the most suitable approach for our research aims. Furthermore, considering the benefits of the bootstrapping procedure, the bootstrap method with 5,000 resamples was employed to estimate the 95% confidence intervals (CI) for the indirect effect (Preacher and Hayes, 2008; Hayes, 2018). All statistical analyses were performed using SPSS version 26.

## Results

Descriptive analyses revealed that the skewness values (ranging from −1.59 to 0.48) and kurtosis values (ranging from −0.76 to 3.65) fell within the acceptable range for normal distributions (see Table 1). Regarding the correlation analysis, perceived occupational stress exhibited significant negative correlations with meaning in life, psychological flexibility, job satisfaction, and job performance. Additionally, it demonstrated a significant positive correlation with psychological distress. Meaning in life and psychological flexibility were significantly positively correlated with both job satisfaction and job performance, while they were significantly negatively correlated with psychological distress. Furthermore, a significant positive correlation was observed between meaning in life and psychological flexibility. A summary of the correlation analysis results can be found in Table 1.

**Table 1:** Descriptive statistics and correlation results.

	Descriptive				Reliability	Correlations					
Variable	Mean	SD	Skewness	Kurtosis	α	1	2	3	4	5	6
1. Perceived occupational stress	12.02	3.95	0.01	−0.76	0.85	1	−0.54^**^	−0.23^**^	0.38^**^	−0.24^**^	−0.15^**^
2. Job satisfaction	18.42	3.94	−0.65	0.17	0.85		1	0.40^**^	−0.38^**^	0.38^**^	0.28^**^
3. Job performance	43.56	6.34	−0.51	1.06	0.91			1	−0.33^**^	0.39^**^	0.50^**^
4. Psychological distress	15.25	3.83	0.48	0.81	0.78				1	−0.38^**^	−0.36^**^
5. Meaning in life	17.76	3.05	−1.59	3.65	0.84					1	0.33^**^
6. Psychological flexibility	21.00	3.27	−0.31	1.14	0.72						1

**p < 0.01.

After conducting preliminary analyses, a mediation analysis was performed to explore the potential mediating role of meaning in life and psychological flexibility in the relationship between perceived occupational stress and job satisfaction, job performance, and psychological distress. The results of the mediation analysis indicated significant direct and indirect effects between the variables (see Table 2). In particular, the analysis revealed that perceived occupational stress had a significant predictive effect on both meaning in life (*β* = −0.24, *p* < 0.001) and psychological flexibility (*β* = −0.15, *p* < 0.001). The perceived occupational stress accounted for 6 and 2% of the variance in meaning in life and psychological flexibility, respectively. Additionally, perceived occupational stress (*β* = −0.47, *p* < 0.001), meaning in life (*β* = 0.22, *p* < 0.001), and psychological flexibility (*β* = 0.13, *p* < 0.001) were found to significantly predict job satisfaction, explaining 38% of the variance in this outcome. Furthermore, perceived occupational stress (*β* = −0.11, *p* < 0.001), meaning in life (*β* = 0.23, *p* < 0.001), and psychological flexibility (*β* = 0.40, *p* < 0.001) had a significant predictive effect on job performance, accounting for 32% of the variance in this construct. Moreover, perceived occupational stress (*β* = 0.29, *p* < 0.001), meaning in life (*β* = −0.22, *p* < 0.001), and psychological flexibility (*β* = −0.24, *p* < 0.001) had a significant predictive effect on psychological distress, explaining 28% of the variance in this outcome variable. Notably, the mediation analysis demonstrated a significant indirect effect of perceived occupational stress on job satisfaction, job performance, and psychological distress through meaning in life and psychological flexibility, as indicated in Table 3. This finding suggests that meaning in life and psychological flexibility serve as mediators in the association between perceived occupational stress and job satisfaction, job performance, and psychological distress. Further details, including standardized indirect effects, can be found in Table 3.

**Table 2:** Unstandardized coefficients for the mediation model.

	Consequent																			
	*M_1_* (Meaning in life)				*M_2_* (Psychological flexibility)				*Y_1_* (Job satisfaction)				*Y_2_* (Job performance)				*Y_3_* (Psychological distress)			
Antecedent	Coeff.	*SE*	*t*	*p*	Coeff.	*SE*	*t*	*p*	Coeff.	*SE*	*t*	*p*	Coeff	*SE*	*t*	*p*	Coeff	*SE*	*t*	*p*
*X* (Perceived occupational stress)	−0.19	0.03	−5.83	<0.001	−0.12	0.03	−3.50	<0.001	−0.47	0.03	−13.46	<0.001	−0.18	0.06	−3.04	<0.001	0.29	0.04	7.89	<0.001
*M_1_* (Meaning in life)	–	–	–	–	–	–	–	–	0.29	0.05	6.11	<0.001	0.48	0.08	6.04	<0.001	−0.28	0.05	−5.74	<0.001
*M_2_* (Psychological flexibility)	–	–	–	–	–	–	–	–	0.16	0.04	3.74	<0.001	0.78	0.07	10.78	<0.001	−0.28	0.04	−6.33	<0.001
Constant	20.00	0.40	49.45	<0.001	22.46	0.44	50.92	<0.001	15.55	1.20	12.93	<0.001	20.73	2.03	10.23	<0.001	22.80	1.25	18.22	<0.001
	*R*^2^ = 0.06				*R*^2^ = 0.02				*R*^2^ = 0.38				*R*^2^ = 0.32				*R*^2^ = 0.28			
	*F* = 33.98; *p* < 0.001				*F* = 12.25; *p* < 0.001				*F* = 110.28; *p* < 0.001				*F* = 84.67; *p* < 0.001				*F* = 72.97; *p* < 0.001			

SE, standard error; Coeff, unstandardized coefficient; X, independent variable; M, mediator variables; Y, outcomes or dependent variables.

**Table 3:** Standardized indirect effects and 95% bias-corrected confidence interval.

Path	Effect	*SE*	Boot LLCI	Boot ULCI
Total indirect effect of perceived occupational stress on job satisfaction	−0.07	0.02	−0.11	−0.04
Perceived occupational stress–>Meaning in life–>Job satisfaction	−0.05	0.01	−0.09	−0.03
Perceived occupational stress–>Psychological flexibility–> Job satisfaction	−0.02	0.01	−0.04	−0.01
Total indirect effect of perceived occupational stress on job performance	−0.19	0.04	−0.27	−0.11
Perceived occupational stress–>Meaning in life–>Job performance	−0.09	0.02	−0.14	−0.05
Perceived occupational stress–>Psychological flexibility–> Job performance	−0.10	0.04	−0.18	−0.03
Total indirect effect of perceived occupational stress on psychological distress	0.09	0.02	0.05	0.13
Perceived occupational stress–>Meaning in life–>Psychological distress	0.05	0.01	0.03	0.09
Perceived occupational stress–>Psychological flexibility–> Psychological distress	0.04	0.01	0.01	0.07

Number of bootstrap samples for percentile bootstrap confidence intervals: 5,000.

## Discussion

This study aims to investigate the mediating role of psychological flexibility and meaning in life in the relationship between occupational stress and job performance, job satisfaction, and psychological distress. Our first hypothesis was occupational stress had a direct effect on psychological flexibility, meaning in life, job performance, job satisfaction and psychological distress. The findings have shown that occupational stress has a significant and negative predictive effect on psychological flexibility, meaning in life, job performance and job satisfaction, and positively predicts the level of psychological distress among teachers. This finding can be said to confirm that occupational stress adversely affects mental health and decreases job-related performance and satisfaction. This result concurs with the research suggesting that psychological distress such as depression and anxiety is triggered by occupational stress, that it has a negative correlation with psychological flexibility and that it reduces job performance and job satisfaction (Liberman et al., 2002; Reed, 2016; Devi and Lahkar, 2021; Aldaiji et al., 2022; Quan et al., 2022). Stress can be said to be a psychological problem which influences performance and satisfaction under any circumstances. When individuals are under stress, they may negatively question the purpose of their lives and their psychological flexibility may decrease, thereby reducing their ability to handle negative feelings. Those who work in a constantly stressful environment may have difficulty in concentrating, employees’ decision-making processes may be adversely affected and their productivity may decrease. Besides, by causing depression symptoms among employees (Anbazhagan et al., 2016), occupational stress may reduce employees’ job satisfaction as well as increase their intention to quit and trigger emotional and professional burnout. In this regard, occupational stress has the potential to adversely affect mental health and contribute to the development of mental health issues and common mental disorders, largely as a result of its detrimental impact on job performance and job satisfaction (Stansfeld and Candy, 2006; Mamom et al., 2023).

Our second hypothesis posited a direct influence of psychological flexibility on job performance, job satisfaction, and psychological distress, while the third hypothesis postulated a direct impact of meaning in life on these same factors. The findings provide empirical support for both the second and third hypotheses. The findings on the third hypothesis have yielded similar results to the second hypothesis. Concerning these findings, it was determined that the high-level and positive meaning in life significantly predicted job performance and job satisfaction in a positive-oriented way and that it significantly predicted psychological distress in a negative-oriented way. Psychological flexibility involves the processes of acceptance, present moment, defusion, self-as-a-content, values, and committed action (Hayes et al., 2006; Kashdan and Rottenberg, 2010; Hayes et al., 2012; Gloster et al., 2017). In this respect, handling and accepting problems encountered in work life with common sense, exhibiting awareness-based behaviors and taking decisive actions will foster job performance and job satisfaction, as well as contribute to the minimization of psychological distress. Ochoa Pacheco et al. (2023) unveiled a significant positive relationship between meaning, which is a dimension of psychological empowerment, and job performance. High levels of psychological flexibility or positive perception of the meaning in life can provide solution-oriented and goal-based planning against the difficulties faced in work life. Thus, such negative symptoms as depression, anxiety and anger can be reduced. By ensuring the effective use of functional coping mechanisms, job performance and job satisfaction can be enhanced.

We have identified as our last and fourth hypothesis that the effect of occupational stress on job performance, job satisfaction and psychological distress is mediated by psychological flexibility and the meaning in life. Recent findings have demonstrated that psychological flexibility and the meaning in life have a mediator role in the relationships between occupational stress and job satisfaction, job performance and psychological distress.

The existing literature has shown that occupational stress has a negative effect on job performance and job satisfaction and that psychological flexibility and meaning in life have positive effects on job performance and job satisfaction. Siu et al. (2020) argued that high levels of positive feelings may reduce the effect of occupational stress, absenteeism and presenteeism. A growing body of literature has also suggested that occupational stressors reduce job performance and job satisfaction (Safarpour et al., 2018; Zang et al., 2022). However, the finding of the current study emphasizing that psychological flexibility and the meaning in life decrease this effect as mediators can be said to be an expected result. A high level of psychological flexibility and a positive perception of meaning toward life are thought to empower psychological resilience in the face of occupational stressors that lead to psychological distress.

## Implication

Several studies have explored the influence of occupational stress on various outcomes such as job satisfaction, job performance, and psychological distress (e.g., Stansfeld and Candy, 2006; Dolan, 2007; Kong et al., 2020). However, these studies typically focused on specific pairs of variables, leaving a gap in our understanding of their simultaneous mediational effects. This study addresses this gap by presenting new evidence about the concurrent mediating roles of meaning in life and psychological flexibility in explaining the relationship between occupational stress and work-related outcomes. Therefore, our research contributes significantly to the existing literature, expanding the available knowledge in this field. Current findings suggest that positive psychological factors, such as psychological flexibility and perceived positive meaning in life under stress at the workplace, have some benefits in terms of increasing job satisfaction while reducing psychological distress. Occupational stress is a mental health problem. It can increase mental and emotional dysfunctions, increasing the likelihood of behavioral problems and psychological distress. The identification of protective factors for the prevention of mental health problems plays a key role in improving the wellbeing and psychological health of individuals who face behavioral problems related to occupational stress. For this reason, psychological flexibility and meaning in life come to the fore as protective elements in preventing the negative effects of occupational stress-related disorders and improving the wellbeing and psychological health of individuals. One of the most important measures to minimize the spread of problems caused by occupational stress is the adoption of a lifestyle that protects mental health. Employees, in this way, will mentally protect themselves against experiences which pose the risk of being adversely psychologically affected.

We recommend that decision-makers on international platforms should put occupational stress on their agenda. While many institutions and organizations organize seminars concerning stress management and coping strategies, their effectiveness in practice remains unclear. The roles of psychological resources like psychological flexibility and meaning in life should be incorporated to deal with occupational stress and its potential consequences on both psychological and work-related outcomes. Also, occupational psychologists or school counselors should plan to provide individual or group support to teachers at the school by cultivating psychological flexibility and meaning in life against occupational stress. Furthermore, managers should create a workplace environment that will make their employees feel valued, even if their employees are not working in the positions they want or their economic expectations cannot be met.

## Limitations

In the present study, some limitations need to be recognized. First of all, it is a cross-sectional study conducted at a single point in time. Experiences regarding occupational stress may develop temporarily depending on the situation or time. In addition, some participants may have low levels of job performance or job satisfaction due to factors other than occupational stress, and some may vary in their psychological flexibility. As a result, it is difficult to come to an unequivocal conclusion about the relationships between variables. We also recommend that a further study should be carried out in which the teaching branches are investigated separately through a longitudinal approach. Second, we used an online approach to reduce paper consumption, save time, increase diversity in the participant population, and collect data more functionally. However, the responses of the participants without internet access or with low levels of digital literacy were underrepresented. In these studies, deepening the examination of the effect of the demographic characteristics of the sample can also help develop intervention strategies to maintain psychological health. It is proposed to design a clinical study. Thus, by encouraging the positive development of psychological flexibility skills and perceived meaning in life (through face-to-face or online methods) while under stress in work life, it will be possible to test whether psychological distress decreases over time and if job performance and job satisfaction also increase. Situations such as the meaning attached to the workplace or a momentary situation creating stress toward work on the day the questionnaire is filled out may be another limitation of the work. Therefore, a study that includes variables such as the position of the employees in the workplace, the meaning they attribute to their occupation, and the presence of a temporary problem they have experienced at the workplace in recent days may expand the limitation in this regard to some extent. This study provides evidence about the relationship between occupational stress, psychological flexibility, perceived meaning in life, job performance, job satisfaction, and psychological distress of teachers in Türkiye. The level of influencing the variables associated with occupational stress may vary according to countries, rural/ metropolitan cities or occupations. This may affect the generalizability. Another limitation of this study is the reliance on self-report measures for data collection, which may be subject to response bias and social desirability effects. Future research could benefit from incorporating objective measures in addition to self-reports to provide a more comprehensive understanding of the variables under investigation. Finally, it is important to note that the current study adopts a cross-sectional research design, which inherently limits the capacity for robust causal inference when compared to longitudinal studies (Maxwell and Cole, 2007). Cross-sectional research, by its nature, includes variations across different temporal, contextual, and spatial dimensions. While this study has explored linear relationships among variables and discussed potential causal linkages, it is essential to acknowledge that the cross-sectional design precludes definitive causal determinations. Therefore, it is important to exercise caution when interpreting the results, especially regarding causal relationships, such as the findings of mediation effects.

## Conclusion

In sum, the present study provides evidence of the relationships between teachers’ occupational stress, psychological flexibility, meaning in life, job performance, job satisfaction and psychological distress. The findings showed that psychological flexibility and meaning in life had mediating effects that reduced the negative effects of occupational stress on job performance, job satisfaction and psychological distress. Occupational stress is thought to influence individuals’ health, productivity, and wellbeing, both globally and locally.

## Data availability statement

The raw data supporting the conclusions of this article will be made available by the authors upon reasonable request.

## Ethics statement

The studies involving humans were approved by the Ethics Committee of Batman University. The studies were conducted in accordance with the local legislation and institutional requirements. The participants provided their written informed consent to participate in this study.

## Author contributions

MY: Writing – review & editing, Writing – original draft, Visualization, Validation, Supervision, Software, Resources, Methodology, Investigation, Funding acquisition, Formal analysis, Data curation, Conceptualization. ÜD: Writing – review & editing, Writing – original draft, Validation, Supervision, Project administration, Methodology, Conceptualization. AM: Writing – review & editing, Writing – original draft, Resources, Project administration, Funding acquisition.
